# A feasibility study of neoadjuvant talazoparib for operable breast cancer patients with a germline *BRCA* mutation demonstrates marked activity

**DOI:** 10.1038/s41523-017-0052-4

**Published:** 2017-12-06

**Authors:** J. K. Litton, M. Scoggins, D. L. Ramirez, R. K. Murthy, G. J. Whitman, K. R. Hess, B. E. Adrada, S. L. Moulder, C. H. Barcenas, V. Valero, J. Schwartz Gomez, E. A. Mittendorf, A. Thompson, T. Helgason, G. B. Mills, H. Piwnica-Worms, B. K. Arun

**Affiliations:** 1 Department of Breast Medical Oncology, Clinical Cancer Genetics, The University of Texas MD Anderson Cancer Center, 1515 Holcombe Boulevard, Houston, TX 77030 USA; 2 Department of Diagnostic Radiology, The University of Texas MD Anderson Cancer Center, 1515 Holcombe Boulevard, Houston, TX 77030 USA; 3 Department of Biostatistics, The University of Texas MD Anderson Cancer Center, 1515 Holcombe Boulevard, Houston, TX 77030 USA; 4 Department of Breast Surgical Oncology, The University of Texas MD Anderson Cancer Center, 1515 Holcombe Boulevard, Houston, TX 77030 USA; 5 Department of Systems Biology, The University of Texas MD Anderson Cancer Center, 1515 Holcombe Boulevard, Houston, TX 77030 USA; 6 Department of Cancer Biology, The University of Texas MD Anderson Cancer Center, 1515 Holcombe Boulevard, Houston, TX 77030 USA

## Abstract

This study was undertaken to determine the feasibility of enrolling breast cancer patients on a single-agent-targeted therapy trial before neoadjuvant chemotherapy. Specifically, we evaluated talazoparib in patients harboring a deleterious BRCA mutation (BRCA+). Patients with a germline BRCA mutation and ≥1 cm, HER2-negative primary tumors were eligible. Study participants underwent a pretreatment biopsy, 2 months of talazoparib, off-study core biopsy, anthracycline, and taxane-based chemotherapy ± carboplatin, followed by surgery. Volumetric changes in tumor size were determined by ultrasound at 1 and 2 months of therapy. Success was defined as 20 patients accrued within 2 years and <33% experienced a grade 4 toxicity. The study was stopped early after 13 patients (*BRCA*1 + *n* = 10; *BRCA*2 + *n* = 3) were accrued within 8 months with no grade 4 toxicities and only one patient requiring dose reduction due to grade 3 neutropenia. The median age was 40 years (range 25–55) and clinical stage included I (*n* = 2), II (*n* = 9), and III (*n* = 2). Most tumors (*n* = 9) were hormone receptor-negative, and one of these was metaplastic. Decreases in tumor volume occurred in all patients following 2 months of talazoparib; the median was 88% (range 30–98%). Common toxicities were neutropenia, anemia, thrombocytopenia, nausea, dizziness, and fatigue. Single-agent-targeted therapy trials are feasible in BRCA+ patients. Given the rapid rate of accrual, profound response and favorable toxicity profile, the feasibility study was modified into a phase II study to determine pathologic complete response rates after 4–6 months of single-agent talazoparib.

## Therapeutics: PARP inhibitor looks promising ahead of surgery

An investigational PARP inhibitor seems safe and possibly effective when given ahead of surgery to women with *BRCA*-mutated breast cancer. Jennifer Litton and colleagues from the University of Texas MD Anderson Cancer Center in Houston, USA, conducted a small feasibility study to see if patients with stage I-III breast cancer and inherited mutations in *BRCA1* or *BRCA2* would put off their standard course of chemotherapy ahead of surgery to first receive two months of talazoparib, an experimental inhibitor of poly ADP ribose polymerase (PARP), an enzyme involved in DNA damage repair. The trial was a resounding success. In fact, owing to rapid patient enrollment, decreases in tumor volume among all 13 participants and few signs of serious side effects, the researchers amended the study protocol to give talazoparib for longer and test for therapeutic efficacy.

## Introduction

The *BRCA* genes were first described in families with breast and ovarian cancers through genetic linkage analysis.^1–3^ A meta-analysis of 10 studies estimated the lifetime risk of breast cancer in *BRCA*1 carriers to be ~47–66 and 40–57% in *BRCA*2 carriers. The ovarian cancer risk was estimated at 35–46% in *BRCA*1 carriers and 13–23% in *BRCA*2 carriers.^4^ Other studies have estimated these risks to be even higher.^5^ Given these risks, targeting BRCA pathogenic mutations in breast cancer patients is an attractive strategy for systemic therapy.

The use of neoadjuvant chemotherapy (NAC) has been an effective tool initially implemented to downstage locally advanced tumors to achieve surgical resection. The use of NAC also provides prognostic information and provides early evidence of drug efficacy. A previous analysis from The University of Texas MD Anderson Cancer Center evaluated response to NAC in women with a known *BRCA* mutation.^6^ Of the *BRCA*1 carriers, 26/57 (46%) achieved a pathologic complete response (pCR). In the multivariate model, both *BRCA*1 and triple receptor-negative breast cancer (TNBC) were independent predictors of pCR. In this reported cohort, >80% of the patients received an anthracycline and taxane-based therapy. Other studies have shown a significant increase in response to platinum-based therapy, but many of these studies combine TNBC patients both with and without a *BRCA* mutation. Silver et al. evaluated 28 women with TNBC who received four cycles of cisplatin at 75 mg/m^2^ every 21 days, with 22% achieving a pCR.^7^ Both of the *BRCA*1 mutation carriers had a pCR in this small cohort. Sikov et al. described an increase in pCR from 41 to 54% in patients with TNBC, unselected for *BRCA* status, with the addition of carboplatin to standard anthracycline and taxane-based chemotherapy.^8^

In addition to using standard chemotherapies for *BRCA* mutation carriers, there is strong rationale for using therapeutics blocking DNA repair mechanisms. Poly-(adenosine diphosphate (ADP)-ribose) polymerase (PARP) is a family of enzymes responsible for multiple cellular processes, including DNA repair through the base excision repair pathway and maintenance of genetic stability.^9^ Prevention of DNA base excision repair (“synthetic lethality” with defects in homologous recombination) and PARP-trapping mechanisms contribute to efficacy in models.^10,11^ PARP inhibitors have been tested in TNBC and other solid tumors, with the most significant single-agent antitumor activity concentrated in patients with known *BRCA* mutations.^12–15^ The lack of homologous recombination due to BRCA mutations coupled with PARP suppression inhibits the recruitment of DNA-repair complexes, leading to stalled replication forks and cell death.^16^

Talazoparib (formerly BMN 673), in particular, is a promising PARP inhibitor, given its significant potency and superior PARP-trapping properties.^10,17^ In the phase I, dose-escalation trial of talazoparib, 14 patients with metastatic breast cancer were treated at the recommended phase 2 dose of 1 mg per day. All patients had a known BRCA mutation and the overall response rate was 50%, with five patients having stable disease for 24 weeks. The clinical benefit rate was therefore 86%.^18^ Given the response and tolerability of talazoparib, phase II (ABRAZO) and phase III (EMBRACA) trials were initiated. Given the strong known and demonstrated relation of PARP inhibition in patients with BRCA-mutated tumors, further information is still warranted on efficacy of therapy in operable breast cancer as well as mechanisms of resistance.

Although there are multiple ongoing clinical trials with PARP inhibitors in both metastatic and early-stage breast cancer, there has not yet been a single-agent evaluation of any PARP inhibitor in the neoadjuvant setting. Combination strategies of PARP inhibitors and chemotherapies are underway; however, overlapping toxicities of cytopenias may limit the ability to give full doses of both PARP inhibitors and/or chemotherapeutics. Concerns have also arisen regarding delaying systemic chemotherapy, especially in younger patients with aggressive tumors, which are often seen in *BRCA* mutation carriers.^19^ Therefore, this feasibility study was undertaken to evaluate whether a potent trapping PARP inhibitor, talazoparib, would be acceptable given alone to early-stage breast cancer patients prior to initiating neoadjuvant systemic chemotherapy. In addition, efficacy and mechanisms of activity and resistance would be able to better be evaluated through correlatives in previously untreated operable breast cancers. Here we report on the trial’s primary objectives of feasibility and toxicity as well as initial efficacy data.

## Results

### Patient demographics

Thirteen patients were accrued to the trial over ~8 months (30 July 2015 to 2 March 2016), demonstrating feasibility. After these, first 13 patients were accrued, and after clinical responses and toxicity were assessed, this trial was halted early as the likelihood of not meeting the trial end points were low. The trial was subsequently amended to allow for longer single-agent treatment of talazoparib (without NAC) prior to surgery to assess pathologic response. Patient demographics are listed in Table 1. The median age was 40 years (range 25–55) and most patients had deleterious mutations in *BRCA1* (*n* = 10; 77%), were triple-negative (*n* = 9; 69%), and/or had stage II disease (*n* = 9; 69%). All patients had invasive ductal carcinoma, with the exception of one patient who had metaplastic breast cancer.

**Table 1:** Patient characteristics

Patient characteristics		
Age (years)	Median (range)	40 (25–55)
Race	White	5
	Black	4
	Hispanic	3
	Asian	1
Clinical T stage	T1	2
	T2	9
	T3	2
Clinical N stage	N0	12
	N1	1
	N2	0
	N3	0
Clinical stage	I	2
	II	9
	III	2
Estrogen receptor	Positive	5
	Negative	8
Progesterone receptor	Positive	3
	Negative	10
*BRCA* (1 or 2)	1	10
	2	3
Histology	Ductal	12
	Metaplastic	1
Grade	1	0
	2	1
	3	12
Compliance	100%	12
	57%	1
Days from last talazoparib to first day of chemotherapy	Median (range)	8 (1–23)*
Chemotherapy regimen used	AC followed or preceded by weekly taxol	13
	Addition of carboplatin to weekly taxol	6
Dose reduction of talazoparib	One patient	25% Dose reduction secondary to cytopenia that occurred 48 days from the initiation of therapy after holding therapy for 7 days and decreased to grade I

Note: the 23 days was at the request of the patient

### Toxicity

The trial initially set out to evaluate whether less than 33% of patients would be able to tolerate talazoparib without a grade 4 toxicity. After the first 13 patients completed 2 months of talazoparib, no grade 4 toxicities were observed. Most of the toxicities observed were related to cytopenias, fatigue, dizziness, and nausea. Table 2 details the highest-grade toxicity observed during the 2 months of talazoparib for each patient. Of note, although there were several grade 3 toxicities, only one led to a dose reduction as the others were observed at the end of the 2 months of therapy. Another patient had difficulty with drug compliance, although that patient still had an excellent clinical response. As this patient had only grade I toxicities, her noncompliance was not attributed to toxicity.

**Table 2:** Patient toxicities

Adverse event	Grade-1	Grade-2	Grade-3	Grade-4
Anemia	6	1	2	
Leukopenia	3	4	1	
Neutropenia (decreased ANC)	2	2	3	
Thrombocytopenia	3	1	1	
Mucositis/mouth sore	4	1		
Dizziness	8			
Fatigue	7			
Nausea	7			
Elevated ALT/AST	4			
Dyspnea	3			
GI disorder (stomach cramps/pain)	3			
Headache	3			
Hyperbilirubinemia	2			
Memory impairment	2			
Hypomagnesemia	2			
Alopecia	2			
Constipation	2			
Dry mouth	1			
Eye redness/pain	1			
Hyperkalemia	1			
Hypokalemia	1			
Myalgia	1			
Nail discoloration	1			
Neuropathy	1			
Rash	1			

^*^Adverse events were monitored from the date of consent to the date on which patients started new treatment

During the conventional chemotherapy treatments, dose delays and dose reductions were required for carboplatin in patients 1, 2, and 6. Patient 3 had the 12th dose of weekly paclitaxel held because of nail changes and patient 7 had a dose reduction of weekly paclitaxel at week 6 because of peripheral neuropathy. All patients were able to receive full dosing of the AC chemotherapy.

### Clinical responses

**Fig. 1:**
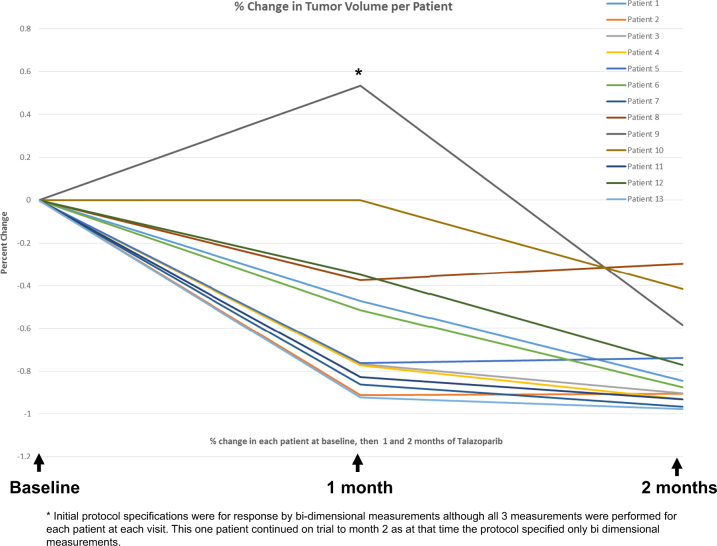
Percent change in tumor volume per patient at 1 month and 2 months of therapy with talazoparib

All 13 patients had a clinical response with a median decrease in tumor volume of 88% (range 30–98%). Table 3 reviews per patient treatment characteristics and percent change in tumor volume as per ultrasound. Figure 1 demonstrates tumor changes per patient across the 2 months of talazoparib administration. In addition, the pathologic response after 2 months of talazoparib and then standard chemotherapy regimen is also reported. Importantly, all patients with TNBC had an residual cancer burden (RCB)-0 or RCB-I at the time of surgery. Three patients had RCB-II disease and all had hormone receptor-positive disease. There were no patients with RCB-III disease at the time of surgery. Patient 9 remained on study as this was prior to the protocol amendment assigning response to tumor volume, and the patient had stable disease by bi-dimensional measurements

**Table 3:** Clinical outcomes

Patient	Clinical stage	Clinical lymph node stage	Biology	*BRCA*	Baseline UTV*	1 Month UTV	2 Month UTV	% UTV change from baseline to 2 months	Chemotherapy	RCB
1	2	0	TNBC	1	0.653	0.346	0.101	−84.5	tc-ac	0
2	2	0	ER+	1	3.041	0.264	0.283	−90.7	tc-ac	II
3	1	0	TNBC	1	1.131	0.264	0.11	−90.3	t-ac	I
4	2	0	ER+	2	8.313	1.885	0.565	−93.2	t-ac	0
5	2	0	ER+	1	5.091	1.204	1.325	−74.0	tc-ac	0
6	2	0	TNBC-metaplastic	2	29.688	14.426	3.695	−87.6	ac-tc	I
7	2	0	TNBC	1	9.739	1.334	0.33	−96.6	ac-t	0
8	3	1	ER+	2	25.871	16.211	18.165	−29.8	t-ac	II
9**	3	0	TNBC	1	3.828	5.875	1.583	−58.6	t-ac	0
10	1	3	TNBC	1	1.131	1.131	0.66	−41.6	tc-ac	0
11	2	0	ER+	1	15.6	2.686	1.068	−93.2	ac-t	II
12	2	0	TNBC	1	3.811	2.488	0.871	−77.1	t-ac	I
13	2	0	TNBC	1	11.362	0.88	0.2544	−97.8	t-ac	0

*UTV ultrasound tumor volume as calculated by (length × width × height × *π*)/6

**Of note, this patient continued on study after the first month as per protocol, the largest bi-dimensional, not tri-dimensional, were used for evaluation, although all three were taken at each time point and recorded. Of note, no patients were PR+ and ER−

*RCB* residual cancer burden

Chemotherapy: *t* taxol, *ac* doxorubicin + cyclophosphamide, *tc* taxol + carboplatin

*ER* estrogen receptor-positive (≥10%), *TNBC* triple-negative breast cancer

### Statistical considerations

As 0/13 patients experienced grade 4 toxicities, the observed grade 4 toxicity rate = 0%; therefore, the posterior probability that the grade 4 toxicity rate > 0.3 = 0.7% and the 90% credible interval = (0, 19%). The study was therefore halted early as there was a small chance that even if continued it would not meet the toxicity success end point. With 13/13 clinical responses, the response rate = 100% and the 90% credible interval = (81, 100%). With 10/13 patients achieving RCB-0/I after talazoparib, systemic chemotherapy, and then definitive surgery, this rate = 77% with 90% credible interval = (53, 99%). Specifically, all of the patients with TNBC achieved RCB-0/I (8/8 = 100%). In contrast, for patients with estrogen receptor-positive breast cancer, the rate was 40% (2/5) with *p* = 0.035 (Fisher exact test).

## Discussion

In this pilot trial, we sought to evaluate the use of the single-agent PARP inhibitor, talazoparib, in a 2-month window prior to initiating standard of care chemotherapy followed by definitive surgery. Initially, this trial was designed as a 20-patient pilot in order to establish that a future neoadjuvant trial would be viable and with acceptable toxicity. However, after 13 patients completed 2 months of talazoparib, there was substantial clinical response and excellent tolerance to therapy. The trial accrued 13 patients in ~8 months. The median tumor volume decreases with 2 months of talazoparib was 88% (range 30–98%), and there were no grade 4 toxicities observed. All patients were able to receive timely standard of care third-generation chemotherapy regimens after talazoparib. Because of the response and favorable toxicity profile, the feasibility portion of this study was closed early and the study expanded to up to 6 months of talazoparib prior to surgery.

In addition to evaluating clinical response after 2 months of talazoparib, pathologic response after subsequent chemotherapy and surgery was also evaluated. RCB is a continuous index combining pathologic measurements of the primary tumor (size and cellularity) and nodal metastases (number and size), and is an independent predictor of distant relapse-free survival.^20^ In TNBC, patients with minimal residual disease (RCB-I) have the same prognosis as patients experiencing pCR (RCB-0) in patients with TNBC. On the other hand, patients with extensive residual disease (RCB-III) have a poor prognosis. In this feasibility study, all patients with TNBC had an RCB-0 or -I at the time of surgery, including the patient with metaplastic breast cancer—a subtype known to have limited response to NAC. All three patients with RCB-II had hormone receptor-positive disease, therefore, less likely to have complete pathologic response to NAC and are receiving adjuvant endocrine therapy.^21^ There were no patients with RCB-III disease identified at the time of surgery.

This study has several important limitations. First, as a feasibility study, there were only 13 patients enrolled; however, the end points of feasibility to accrue and toxicity were favorable and indicated that the goals set for feasibility in a 20-patient study would have been met. Second, ultrasound was used to determine response to single-agent talazoparib rather than pathologic response, which is considered the gold standard in evaluating therapeutic effect in the neoadjuvant setting. This pre-NAC design was necessary to establish efficacy and patient safety in a potentially curable group of patients. Although a 2-month therapeutic trial with a targeted agent that demonstrates a substantial volumetric reduction in the primary tumor cannot be used to estimate rates of pCR, we believe that these findings do justify a clinical trial to determine the pCR rates of talazoparib as monotherapy over a longer period of time (4–6 months) for the treatment of BRCA + patients with localized breast cancer. In addition, further monitoring for toxicity is needed and results from ongoing clinical trials with talazoparib for metastatic patients will add to the available toxicity data.

Ultrasounds were used to evaluate response during the course of the trial due to the required biopsies, cost, patient comfort, and Institutional practice. While no imaging may take the place of histologic assessment at this time, magnetic resonance imaging (MRI) is also an accepted imaging tool for assessing response to neoadjuvant therapy. Although many studies suggest that MRI is the more reliable imaging modality to assess treatment response, overestimation and underestimation of residual disease are known drawbacks of breast MRI in patients receiving NAC. A recent study showed similar agreement for MRI and US with post-chemotherapy pathological tumor size.^22^ However, this agreement varies with breast cancer subtype. Breast ultrasound does have the benefit of decreased cost, which would have been prohibitive in this trial in addition to potentially increasing discomfort to the patient.^23,24^ Candelaria et al. demonstrated that measurement of tumor volume on ultrasound at mid treatment has a good correlation with RCB, specifically in TNBC and HR + /HER2− tumors.^25^ In addition, as part of the ultrasound evaluation, a biopsy was done for future correlative science, which was easier and directly visualized through ultrasound. Breast ultrasounds have also been used for evaluation during larger neoadjuvant trials for evaluation of response during the course of therapy.^26,27^ It is important to note, however, that the degree of response as seen clinically by exam and ultrasound exceeded our expectations for a single agent in this setting and for which we have no historical controls to a single-agent-targeted therapy. This led to stopping this portion of the trial and moving to the next trial where we will be able to more accurately assess pathologic response to 4–6 months of single-agent talazoparib.

As all 13 patients had responses, many of which were extensive, this feasibility trial validates a strategy to move efficacious agents with a paired biomarker, in this case a known *BRCA* pathogenic variant, quickly into an early-stage breast cancer clinical trial. As we continue to explore new agents and add to the standard of care backbone of anthracycline and taxane-based chemotherapies, there have been some incremental improvements, but often at the cost of extra toxicities. In addition to investigating adding therapies to the chemotherapy backbone, there is also a need for investigating reducing therapies and potentially sparing patients from toxicity while still providing curative therapy.

Given the findings of this window trial, an expansion cohort has been initiated. This expansion is planned to accrue 20 patients to receive at least 4 and up to 6 months of therapy with single-agent talazoparib prior to definitive local therapy. Baseline correlative studies and every 2 month ultrasounds will continue while on talazoparib. Additional biopsies will be obtained should progression be identified in order to evaluate mechanisms of resistance. Because patients will undergo surgery immediately following talazoparib therapy, this expansion will provide a pathologic assessment of neoadjuvant talazoparib.

## Conclusions

Talazoparib was able to be given for 2 months prior to NAC in patients with a known *BRCA* 1 or 2 deleterious pathogenic variant without significant toxicity or inability to safely administer NAC. All 13 patients had a clinical response to neoadjuvant talazoparib justifying a neoadjuvant expansion trial.

## Methods

The primary objective of this study was to evaluate the feasibility of accruing patients to a trial using talazoparib prior to initiating standard neoadjuvant therapies. The trial was designed to evaluate whether patients would accept a delay in chemotherapy and whether 20 patients would accrue within 2 years at a single center. Secondary objectives included toxicity evaluation of 2 months of talazoparib in the neoadjuvant setting and the ability to follow with standard chemotherapy regimens. Aggregate toxicity rate was followed after seven patients were enrolled. If greater than 33% of the patients enrolled had either a grade 4 toxicity attributable to the treatment, or required a delay in treatment for greater than 4 weeks due to toxicity, the study would have been discontinued. Patients were identified for this trial if they had a *BRCA*1 or *BRCA*2 pathogenic variant as identified by a CLIA-certified laboratory and presented with operable breast cancer (stages I–III). Additional inclusion criteria included: ≥1 cm primary tumor; any hormone receptor status but HER2 non-amplified or 0 or 1 by immunohistochemistry as per the current ASCO-CAP guidelines;^28^ no previous surgery, radiation, or systemic therapy for breast cancer except prior surgery for ductal carcinoma in situ was allowed; negative pregnancy test and the patient was considered medically fit to undergo NAC by the treating oncologist. Patients needed to be at least 5 years from the treatment of a previous non-breast malignancy and were able to take oral medications. This trial was performed after approval by the Institutional Review Board and trial identifier is NCT02282345. Data were anonymized so as to protect the identities of subjects involved in the research. A written, informed consent was obtained from each participant. The data that support the findings of this study are available from the corresponding author upon reasonable request.

Talazoparib was orally administered at a dose of 1 mg per day for 2 months prior to starting the taxane and anthracycline chemotherapy regimen of the physician’s choice. This regimen could also include the addition of carboplatin.^8^ Breast ultrasounds, including evaluation of the regional nodal basins, were performed by fellowship-trained breast imagers at baseline, at 1 month after initiation of therapy and at the completion of 2 months of therapy. Initially patients were assessed for response by bi-dimensional ultrasound measurements, although 3-dimensional measurements were always prospectively documented. Therefore, patient 9 remained on study as by bi-dimensional, the tumor was stable. T-methoidhe protocol was subsequently amended to utilize all three provided measurements for further evaluation. The protocol was amended to evaluate response by tumor volume. Tumor volume was estimated using 3-dimensional measurements of the primary tumor (length × width × height × *π*)/6.^29^ Clinical response was considered to be at least a 20% decrease in the volume of the primary breast tumor. Final pathology was evaluated using the RCB score and pCR/RCB-0 was defined as no invasive disease in breast and no disease in the lymph nodes.^20^ Assessment of residual cancer burden calculations can be accessed online at www.mdanderson.org/breastcancer_RCB.

Toxicities were monitored and documented as per the Common Terminology Criteria for Adverse Events version 4.03. Toxicities were reported with the highest grade observed per individual. Adverse events at baseline or otherwise determined to be unrelated to the study drug were not reported in the toxicity assessment for this analysis. Dose reductions were made for a grade 3 or 4 toxicity as per protocol. The study drug was held until the toxicity resumed to a grade 1 and then decreased by 0.25 mg/day.

### Ethics Statement

This trial was conducted under an Institutional Review Board approved protocol 2014-0045 and in accordance with relevant guidelines at The University of Texas MD Anderson Cancer Center.
